# The FOXP1-ABCG2 axis promotes the proliferation of cancer stem cells and induces chemoresistance in pancreatic cancer

**DOI:** 10.1038/s41417-025-00896-7

**Published:** 2025-04-01

**Authors:** Woosol Chris Hong, Minsoo Kim, Ju Hyun Kim, Hyeon Woong Kang, Sungsoon Fang, Hye-Sol Jung, Wooil Kwon, Jin-Young Jang, Hyo Jung Kim, Joon Seong Park

**Affiliations:** 1 Department of Medicine, Yonsei University College of Medicine, Seoul, Republic of Korea; 2 Korea Brain Korea 21 PLUS Project for Medical Science, Yonsei University, College of Medicine, Seoul, Republic of Korea; 3 Department of Surgery and Cancer Research Institute, Seoul National University College of Medicine, Seoul, Republic of Korea; 4 Biomedical Research Institute, Seoul National University Hospital, Seoul, Republic of Korea

**Keywords:** Tumour biomarkers, Cancer stem cells

## Abstract

Pancreatic cancer is an aggressive disease with low survival and high recurrence rates. A major obstacle in treating pancreatic cancer is the frequent development of chemoresistance to the standard therapeutic drug, gemcitabine. One mechanism by which pancreatic cancer develops chemoresistance is through the proliferation of cancer stem cells (CSC). However, the mechanisms regulating stemness in chemoresistant tumors remain unclear. Here, we found that the expression of the transcription factor Forkhead Box P1 (FOXP1) was elevated in chemoresistant pancreatic cancer and crucial for establishing CSC characteristics. Silencing FOXP1 reduced the expressions of stemness-associated genes and diminished the formation of both spheroids and colonies, highlighting the crucial role of FOXP1 in regulating stemness in chemoresistant tumor cells. Mechanistically, we discovered that FOXP1 regulates the expression of ATP-binding cassette superfamily G member 2 (ABCG2), which induces the efflux of gemcitabine. Knockdown of FOXP1 reduced the expression of ABCG2, resulting in decreased proliferation and increased sensitivity to gemcitabine. Moreover, the inhibition of FOXP1 in orthotopic mouse models reduced tumor growth and proliferation, and enhanced sensitivity to gemcitabine. Together, our data reveal FOXP1 as a potent oncogene that promotes CSC growth in chemoresistant pancreatic cancer.

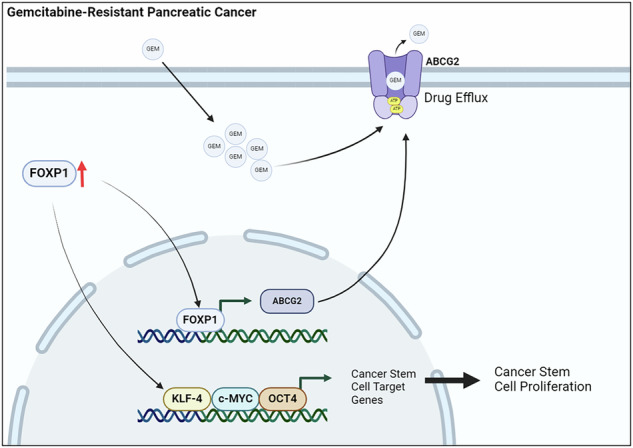

## Introduction

Pancreatic ductal adenocarcinoma (PDAC) is an aggressive and malignant form of pancreatic cancer, with a 5-year overall survival rate of only 13%, one of the lowest rates among solid cancers [1]. Gemcitabine is used as a primary chemotherapeutic agent for PDAC; however, tumors often develop drug resistance, which leads to recurrence [2, 3]. The specific cellular processes and key regulators that contribute to gemcitabine resistance remain poorly understood [4].

Recent studies have highlighted the impact of tumor heterogeneity on the development of chemoresistance [5, 6]. Tumor heterogeneity refers to the genetic and phenotypic diversity of cancer cells within a tumor, as the tumor microenvironment comprises numerous cell types [7, 8]. Cancer stem cells (CSCs) are a sub-population of cells that drive tumor progression and relapse [9]. CSCs exhibit stem cell-like characteristics and play key tumorigenic roles by driving tumor progression, metastasis, and drug resistance [10]. In pancreatic cancer, CSCs are highly plastic with frequent metabolic alterations and promote resistance to chemotherapy and immunotherapy [11, 12]. Therefore, developing biomarkers to identify genes that mediate CSC proliferation is essential. CSCs are identified by a host of cell surface antigens, such as CD133, CD24, EpCAM, and Lgr5, as well as Yamanaka Factors, such as OCT4, Sox2, c-MYC, and KLF4 [13, 14]. However, CSC biomarkers directly related to chemoresistance remain unknown.

Forkhead Box protein 1 is one of the four members of subfamily P of the FOX transcription factor family. FOXP1, along with other members of the *FOX* gene family, is well established for its role in development, differentiation, and stem cell maintenance [15]. Recently, FOXP1 was found to play a crucial oncogenic role in several cancers. FOXP1 drove tumor progressions in osteosarcoma and bladder cancer [15, 16]. In addition, the inhibition of FOXP1 in ovarian cancer decreased CSC characteristics [17]. In pancreatic cancer, FOXP1 has both tumor-suppressive and oncogenic roles [18, 19]. However, neither the association between FOXP1 and chemoresistance nor the link to CSC proliferation in pancreatic cancer has been reported.

In this study, we observed that FOXP1 is highly upregulated in gemcitabine-resistant (GR) pancreatic cancer and that higher FOXP1 expression leads to unfavorable patient outcomes. The inhibition of FOXP1 led to a significant decrease in the CSC-like characteristics of GR PDAC cells. FOXP1 depletion also led to metabolic reprogramming in GR cells by enhancing glycolysis and lactate secretion, which reinforced chemoresistance. We also found that FOXP1 directly modulated the expression of ABCG2, a membrane transporter that promotes the efflux of gemcitabine from cancer cells. Collectively, our findings suggest that FOXP1 is a potential target for combating CSC proliferation in chemoresistant pancreatic cancer.

## Materials and methods

### Patient tissue collection

This study adhered to the ethical principles outlined in the Declaration of Helsinki and was approved by the Institutional Review Board (IRB) of the Gangnam Severance Hospital (IRB No.3-2021-0414). Tissue samples were collected from patients with PDAC and individuals with normal pancreatic tissue who underwent surgical resection or biopsy at Gangnam Severance Hospital.

### Patient selection

Patients diagnosed with PDAC at Gangnam Severance Hospital (2018–2019) underwent pancreatic resection followed by adjuvant gemcitabine chemotherapy (1000 mg/m^2^ over 30 min weekly for 3 weeks in a 4-week cycle, totaling six cycles). Patients without recurrence within 6 months post-chemotherapy were classified as gemcitabine-sensitive (GS), while those with recurrence were considered gemcitabine-resistant (GR). Written informed consent was obtained from all participants before their inclusion in the study.

### Cell culture

Cells were cultured as previously described [7]. Human pancreatic cancer cell lines AsPC-1, BxPC-3, Capan-1, PANC-1, and MIA PaCa-2 were obtained from the ATCC (Manassas, VA, USA). Cells were maintained either in Roswell Park Memorial Institute medium 1640 (RPMI-1640) (Biowest, Riverside, MO, USA) or in Dulbecco’s Modified Eagle’s Medium (DMEM) (Biowest). Both media types were supplemented with 10% fetal bovine serum (FBS; Biowest) and 1% antibiotic-antimycotic (Gibco, Waltham, MA, USA), and cells were incubated at 37 °C in 5% CO_₂_. Capan-1 cells were cultured in a 10-cm dish (SPL Life Sciences, Pocheon, South Korea) in the RPMI medium until they reached 80% confluency before subculture every 7 days. GR cell lines were developed by gradually exposing parental cell lines to increasing gemcitabine (Yuhan, Seoul, Korea) concentrations over 3 months, after which the GR cells were cultured continuously in medium containing 0.5 μM gemcitabine.

### WST-1 cell proliferation

The WST-1 assay was performed as previously described [7]. Cells were seeded in 96-well plates at a density of 5 × 10^3^ cells per well, transfected with siRNA, and treated with gemcitabine for 72 h. Cell viability was assessed using a 10% water-soluble tetrazolium-1 (WST-1) reagent (DoGenBio, catalog code [EZ-500], Seoul, Korea). The absorbance was measured at 450 nm using a VersaMax microplate reader (Molecular Devices, San Jose, CA, USA). IC_50_ values were calculated using GraphPad Prism software (GraphPad Software, Boston, MA, USA).

### Real-time PCR

Total RNA was extracted from cells and tissue samples as previously described [7] using TRIzol reagent (Invitrogen, catalog code [1559626], Waltham, MA, USA) following the manufacturer’s protocol. The extracted RNA was quantified by ND-2000 Spectrophotometer (Thermo Fisher Scientific Inc., Wilmington, DE, USA). qRT-PCR was performed using the Power SYBR Green PCR Master Mix (Applied Biosystems, Waltham, MA, USA) in a 7500 Real-Time PCR System (Applied Biosystems). Relative gene expression was normalized to GAPDH and calculated using the 2^−ΔΔCt^ method.

### RNA sequencing

Total RNA was extracted from cells and tissue samples using TRIzol reagent (Invitrogen) according to the manufacturer’s protocol. Libraries were prepared using the QuantSeq 3′ mRNA-Seq Library Prep Kit FWD (Lexogen, Vienna, Austria). Briefly, an oligo-dT primer with an Illumina-compatible sequence was hybridized to the RNA, followed by second-strand synthesis initiated with an Illumina-compatible linker. The resulting libraries were amplified via adapter addition and then subjected to high-throughput single-end 75-bp sequencing on a NextSeq 550 platform (Illumina Inc., San Diego, CA, USA). Pearson’s correlation analysis was conducted on the RNA expression data, considering correlation coefficients > 0.5 and *P* values < 0.05 as significant. Gene Ontology (GO) and Kyoto Encyclopedia of Genes and Genomes (KEGG) pathway analyses were carried out using the DAVID Bioinformatics Resources 6.8 (https://davidbioinformatics.nih.gov/) with all downstream analyses performed in R version 3.5.1 (R Foundation for Statistical Computing, Vienna, Austria).

### Small-interfering RNA (siRNA) transfection

siRNA-mediated knockdown was performed as previously described [2, 7]. siRNA targeting FOXP1 (FOXP1 siRNA, catalog code [sc-44583], Santa Cruz Biotechnology, Dallas, TX, USA) was dissolved and diluted in RNase-free H₂O. Transfection was performed using Lipofectamine RNAiMAX (Invitrogen, Paisley, UK) following the manufacturer’s protocol. Cells were harvested 48 h post-transfection for further analysis.

### Immunoblotting

Protein lysates were prepared using the radioimmunoprecipitation assay (RIPA) lysis buffer (Rockland, catalog code [MB-030-0050], Philadelphia, PA, USA) and the concentrations were quantified using the Bio-Rad Protein Assay Kit (Bio-Rad, catalog code [5000006], Hercules, CA, USA) according to the manufacturer’s instructions. Equal amounts of lysates were separated on 8–15% SDS-PAGE gels, then transferred onto polyvinylidene fluoride (PVDF) membranes (MiliporeSigma, catalog code [IPVH00010], Burlington, MA, USA). Membranes were blocked and incubated overnight at 4 °C with primary antibodies (1:1000 dilution), followed by HRP-conjugated secondary antibodies (1:5000 dilution). Immunoreactive signals were developed using Clarity Western ECL Substrate (Bio-Rad, catalog code [1705061]) and visualized using the ImageQuant LAS 4000 system (GE Healthcare, Chicago, IL, USA). The primary antibody information is included in Supplementary Table 1.

### Ki-67 staining

Capan-1 cells were fixed in 4% paraformaldehyde (GeneAll Biotechnology, catalog code [SM-P01-050], Seoul, South Korea) for 15 min, permeabilized, and blocked with 5% bovine serum albumin (BSA) (MiliporeSigma, catalog code [A9418-50G]) for 1 h. Samples were incubated with anti-Ki-67 primary antibody (1:200) (Abcam, catalog code [ab15580], Cambridge, UK) at 4 °C overnight, followed by incubation with an Alexa Fluor 488-conjugated secondary antibody (1:500) (Thermo Fisher, catalog code [A-11070]) for 1 h. Nuclei were counterstained with DAPI, and fluorescence images were captured using TCS SP8 STED CW confocal laser scanning microscope (Leica Microsystems, Wetzlar, Germany).

### Wound healing

Wound healing assay was performed as previously described [7]. Briefly, Capan-1 cells were plated in 12-well plates at 2 × 10^5^ cells per well, followed by transfection with siRNA for 48 h. Linear wounds were created using a 10 µL pipette tip. Microscopic images were captured at 0, 16, and 24 h, and the wound area was quantified using the ImageJ software (Version 1.53 T) (NIH, Bethesda, MD, USA).

### Transwell invasion

The invasion assay was performed as previously described [7]. Capan-1 cells were seeded in six-well plates and transfected with siRNA for 48 h. An 8-µm pore Transwell system (Corning Inc., catalog code [3464], Corning, NY, USA) was coated with Matrigel (1:50; Corning) for 1 h. Transfected cells (2 × 10^5^) were seeded on the apical side of the Transwell insert (24-well) in serum-free medium, and medium containing 10% FBS was added to the basal compartment. The cells were incubated for 24 h, then the invaded cells were counted using ImageJ (Version 1.53t).

### Flow cytometry

Flow cytometry (FACS) was performed as previously described [10]. Capan-1 cells were plated in 6-well plates at 5 × 10^5^ cells per well, followed by transfection with siRNA for 48 h. Cells were stained with fluorochrome-conjugated anti-human FITC-CD44 and APC-CD24 antibodies (BioLegend, San Diego, CA, USA) (antibody information included in Primary Antibody Table). The stained cells were analyzed by BD FACSCanto II Cell Analyzer (BD Biosciences, Franklin Lakes, NJ, USA), and data analysis was performed using FlowJo software (Version 10) (BD Biosciences).

### Sphere formation

Capan-1 cells were seeded in ultra-low-attachment 96-well plates (Corning, catalog code [3474]) at a density of 6000 cells per well in serum-free DMEM/F-12 medium (Gibco) supplemented with B27 (1X, catalog code [17504044], Gibco), 20 ng/mL EGF (PeproTech, catalog code [AF-100-15-100 UG], Rocky Hill, NJ, USA), and 20 ng/mL bFGF (PeproTech, catalog code [100-18B-50UG]). The cells were incubated at 37 °C with 5% CO_2_. Spheres were allowed to form for 7–14 days, after which the number and diameter of spheres (>50 µm) were assessed using a light microscope.

### Colony formation

Capan-1 cells were seeded in six-well plates at a density of 500 cells per well and cultured in complete growth medium at 37 °C with 5% CO_2_ for 10 days to allow colony formation. Once the colonies were visible, they were fixed using methanol and stained with 0.5% crystal violet (Sigma-Aldrich, catalog code [C6158-50G]) for 20 min. Colonies were counted and imaged under a light microscope.

### Glucose uptake

The 2-NBDG uptake assay was performed according to the manufacturer’s instructions (Cayman Chemical, catalog code [600470], Ann Arbor, MI, USA). Cells were seeded for 48 h, then incubated with the 2-NBDG reagent and glucose uptake enhancer in the medium for 30 min. The absorbance was measured at 450 nm using VersaMax Microplate Reader (Molecular Devices).

### Lactate secretion

Lactate secretion assay was conducted according to the manufacturer’s protocol (Dogen, catalog code [DG-LAC100]). The cells were treated with assay buffer, homogenized, and mixed with the reaction solution. The mixture was dispensed into a 96-well plate and incubated at 20–25 °C in the dark for 30 min, followed by gentle shaking. The absorbance was measured at 570 nm using Versamax Microplate Reader (Molecular Devices).

### Oxygen consumption rate (OCR) and extracellular acidification rate (ECAR)

OCR and ECAR were measured using a Seahorse XF24 extracellular flux analyzer (Seahorse Bioscience, MA, USA) as previously described [20]. Capan-1 cells were seeded in XF-96 plates, incubated for 24 h, treated with siFOXP1, and then incubated in XF assay media for 1 h at 37 °C without CO_2_. Sequential additions of 1 μM oligomycin, 2 μM FCCP, and 0.5 μM rotenone/antimycin A were performed. ECAR was measured under the same conditions to assess glycolytic activity. The data was normalized to cell counts (per 1000 cells).

### Plasmid DNA transfection

HEK293 cells were seeded in six-well plates and allowed to reach 70–80% confluence at the time of transfection. The WT-ABCG2 and MT-ABCG2 promoter plasmids [17] were mixed with Lipofectamine 3000 reagent (Invitrogen, catalog code [L3000015]) and P3000 reagent in Opti-MEM (Gibco, catalog code [31985070]) according to the manufacturer’s protocol. The DNA-Lipofectamine complex was added to the cells and incubated for 48 h under standard culture conditions. Transfection efficiency was confirmed by downstream analysis.

### Luciferase reporter assay

Cells were transfected with a luciferase reporter plasmid and incubated for 48 h. After treatment, cells were lysed, and luciferase activity was measured using the Dual-Luciferase® Reporter Assay System (Promega, catalog code [E1910], Madison, WI, USA) following the manufacturer’s instructions. Luminescence was quantified using Epoch2 microplate reader (Agilent Technologies, Santa Clara, CA, USA), and the results were normalized to Renilla luciferase activity to control transfection efficiency.

### Chromatin immunoprecipitation (ChIP)

The ChIP assay was performed as previously described [10] using the SimpleChIP Enzymatic Chromatin IP Kit (Cell Signaling Technology, catalog code [9003], Danvers, MA, USA). HEK293 cells were plated in 10-cm plates at 4 × 10^6^ cells per plate, followed by transfection with WT- or MT- ABCG2 plasmid for 48 h. The cells then were cross-linked with 1% formaldehyde and sonicated to yield DNA fragments. Chromatin was immunoprecipitated using anti-IgG and anti-FOXP1 antibodies. Following reverse cross-linking and DNA purification, DNA from the input (1:100 dilution) and the immunoprecipitated samples were analyzed by qPCR (Applied Biosystems).

### Orthotopic mouse model

Six-week-old male nude BALB/c mice (Orient Bio, Seongnam, South Korea) were used. Capan-1 GS and GR cells (3 × 10⁶) were suspended in PBS with Matrigel (Corning) and injected into the pancreas. Tumor growth was monitored for 1 month and FOXP1 Human shRNA lentiviral particles (Origene, catalog code [TL304471V], Rockville, MD, USA) were intravenously administered. One week later, gemcitabine (10 mg/kg) and KO143 (10 mg/kg) (Sigma-Aldrich, catalog code [K2144]) were intraperitoneally injected, 3 times per week. All procedures were approved by the Institutional Animal Care and IACUC of the Seoul Yonsei Pharmaceutical University Experimental Animal Center (approval #2022-0061)

### IHC staining

IHC staining and immunofluorescence were performed as previously described [7]. Poly-L-lysine coated slides were deparaffinized, rehydrated through graded ethanol, and heated in citrate buffer (pH 6) for 30 min for antigen retrieval. After blocking with 10% normal goat serum (Jackson ImmunoResearch, catalog code [005-000-121], West Grove, PA, USA) in PBS for 1 h, slides were incubated overnight at 4 °C with primary antibodies. After incubation with secondary antibodies (Thermo Fisher, catalog codes [A-11070, A-11001]), slides were mounted with Fluoroshield Mounting Medium containing DAPI (Abcam, catalog code [ab104139]) and imaged using a TCS SP8 STED confocal laser scanning microscope (Leica Microsystems).

### Statistical analysis

Statistical analyses were conducted using one-way or two-way analysis of variance using GraphPad Prism (version 8.0; GraphPad Software). Data are expressed as mean ± standard deviation. Significance was set as *p* < 0.05.

## Results

### FOXP1 is upregulated in GR PDAC

**Fig. 1:**
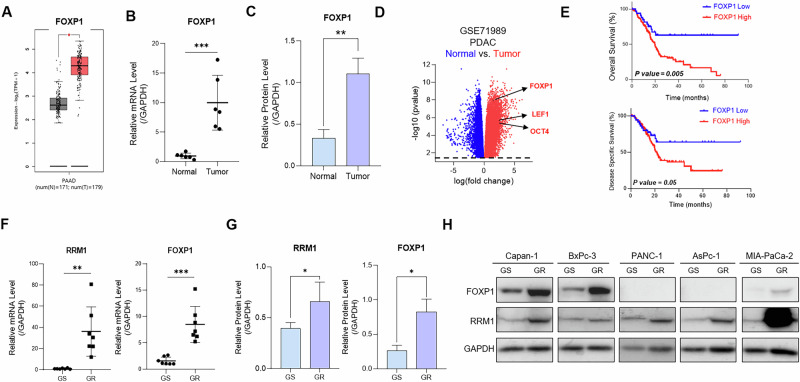
FOXP1 is upregulated in gemcitabine-resistant (GR) PDAC. **A** FOXP1 expression levels in PAAD tumor tissues and adjacent normal tissues. FOXP1 is significantly upregulated in tumor tissues. **B**, **C** mRNA and protein expression analyses reveal higher FOXP1 expression in PDAC tissue samples than in normal tissues. **D** Differential gene expression analysis from the GSE71989 dataset reveals high expressions of FOXP1 and CSC markers LEF1 and OCT4 in tumor tissues [21]. **E** Kaplan–Meier OS and DSS curves for TCGA–pancreatic adenocarcinoma (PAAD) (**F**) mRNA expression levels of FOXP1 and RRM1, a marker of gemcitabine resistance, in GS and GR tissue samples. **G** FOXP1 and RRM1 protein expression levels in GR tissues. **H**, **I** FOXP1 and RRM1 mRNA and protein expression across GS and GR PDAC cell lines, highlighting higher FOXP1 levels in GR cells, with the highest upregulation in Capan-1. *p < 0.05, **p < 0.01, ***p < 0.0001.

We first examined the expression of FOXP1 in pancreatic adenocarcinoma (PAAD) using The Cancer Genome Atlas (TCGA) data from the GEPIA2 dataset. We found that FOXP1 was upregulated in tumor tissues compared to adjacent normal tissues (Fig. 1A). We analyzed FOXP1 expression in tissue samples from patients with PDAC. FOXP1 was upregulated at both the mRNA and protein levels (Fig. 1B, C). FOXP1 was also expressed in PDAC cell lines (Fig. S1A). We then analyzed differential gene expressions in the patient dataset from the GSE71989 and the GSE28735 datasets [21, 22]. FOXP1 was highly expressed in tumor tissues, as well as *LEF1* and *OCT4*, which are markers of CSCs (Fig. S1B, C). Furthermore, analysis of FOXP1 expressions across cancer types from the GEPIA2 and Human Protein Atlas datasets showed that FOXP1 was overexpressed in multiple cancer types, with PAAD displaying the most pronounced upregulation (Fig. S1C). Analysis of overall survival and disease-specific survival in the TCGA-PAAD dataset revealed that patients with high FOXP1 expression had significantly lower survival rates, signifying that FOXP1 is a prognostic marker in pancreatic cancer (Fig. 1E). Next, we assessed the FOXP1 expression in chemoresistant pancreatic cancer. TCGA-PAAD analysis showed that FOXP1 and RRM1, which is a well-established marker upregulated in GR pancreatic cancer [2, 23], have a significantly strong positive correlation (Fig. S1D). The mRNA and protein expression levels of FOXP1 and RRM1 were also increased in tissue samples from patients with GR PDAC (Fig. S1E, F, G). We then established GR cell lines using five PDAC cell lines (Capan-1, BxPc-3, PANC-1, AsPc-1, and MIA-PaCa-2) and increasing doses of gemcitabine (Fig. S1F). Analysis of FOXP1 expression across cell lines confirmed the upregulation of FOXP1 in GR cells compared to that in GS cells (Fig. 1H). To explore the role of FOXP1 in GR cells, we chose Capan-1, which showed the highest rate of upregulation, as the main cell line. Our data show that FOXP1 is an oncogene that is upregulated in chemoresistant PDAC, confirming previous reports on the tumorigenic roles of FOXP1.

### FOXP1 promotes the proliferation of CSCs in chemoresistant PDAC

**Fig. 2:**
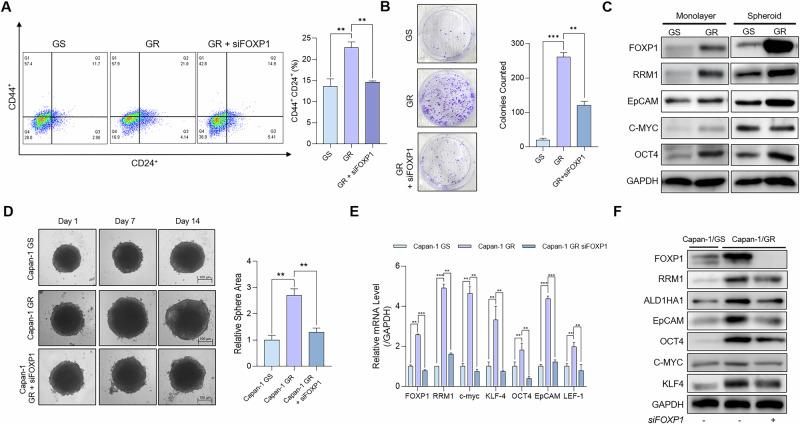
FOXP1 promotes the proliferation of CSCs in GR PDAC. **A** Flow cytometry (FACS) analysis of CD44+ CD24+ double-positive cells in gemcitabine-sensitive (GS), gemcitabine-resistant (GR), and FOXP1 knockdown (KD) GR Capan-1 cells. (**B**) Colony formation assay comparing GS, GR, and GR FOXP1 KD groups. **C** Western blot analysis of CSC marker genes in monolayer and 3D spheroid cultures of GS and GR Capan-1 cells. **D** Spheroid formation over 14 days in GS, GR, and GR FOXP1 KD cells. **E** Quantitative analysis of mRNA levels of CSC markers in GS, GR, and GR FOXP1 KD cells via qRT-PCR. **F** Western blot of CSC markers in Capan-1 GS and GR cells with and without FOXP1 knockdown. Data are represented as the mean ± S.D. of three independent experiments (*n* = 3). Statistical analysis was conducted using one-way or two-way ANOVA, followed by Tukey’s multiple-comparison test. **p < 0.01, ***p < 0.0001.

To investigate the role of FOXP1 in chemoresistant PDAC, we first analyzed the TCGA-PAAD dataset by dividing differential gene expressions by high- and low-FOXP1 expressions. The analysis revealed the expression of *OCT4* was increased in patients with high FOXP1 expression, signifying that FOXP1 plays a role in upregulating CSC-like characteristics in pancreatic cancer (Fig. S2A). STRING protein-protein interaction analysis also revealed that FOXP1 is closely linked to CSC marker genes, further confirming the positive correlation between FOXP1 and CSCs (Fig. S2B). To determine the effects of FOXP1 inhibition on the regulation of CSCs, we knocked down (KD) FOXP1 in GR Capan-1 cells (Fig. S2C). We then analyzed changes in the percentages of CD44^+^ and CD24^+^ cells using flow cytometry (FACS) [10]. FACS analysis showed that the inhibition of FOXP1 led to a marked decrease in the percentage of CD44^+^CD24^+^ double-positive cells in the KD group (Fig. 2A). We also performed a colony formation assay that showed a decrease in the proliferation of CSC-like cells in the KD group, suggesting that FOXP1 plays a role in the growth of CSCs in GR PDAC (Fig. 2B). We then cultured epithelial monolayers and 3D spheroid Capan-1 cells and compared their gene expression levels. GR cells had higher expression of CSC marker genes in both epithelial and spheroid cells, while GR spheroid cells had higher overall gene expression and a more significant increase in FOXP1 and CSC gene expression (Fig. 2C). Therefore, FOXP1 plays a more significant role in resistant CSC than in epithelial cells. To validate the effect of FOXP1 on CSC proliferation, we KD FOXP1 in GR spheroids (Fig. 2D). Consistent with changes in gene expression, we observed a clear reduction in spheroid size after FOXP1 inhibition. In addition, FOXP1 depletion decreased the expression of prominent CSC markers (Fig. 2E, F), leading us to conclude that FOXP1 plays a crucial role in the proliferation and maintenance of chemoresistant CSCs.

### FOXP1 enhances epithelial-to-mesenchymal transition and induces proliferation in GR PDAC

**Fig. 3:**
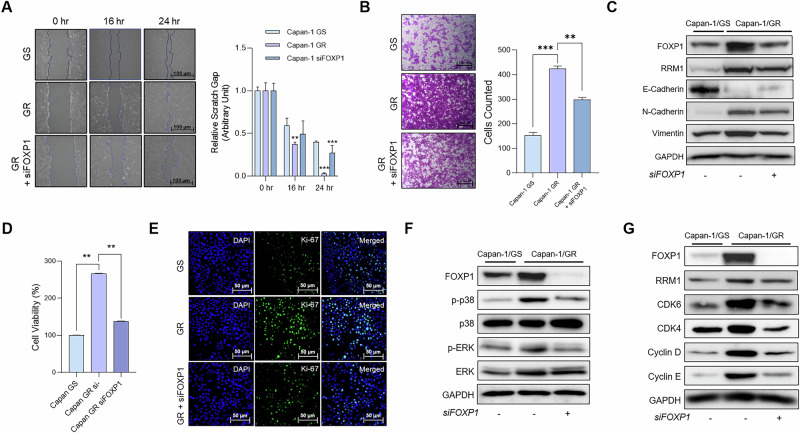
FOXP1 enhances EMT and induces proliferation in GR PDAC. **A** Wound healing assay of GS, GR, and KD Capan-1 cells at 0, 16, and 24 h. Scale bar = 100 μm (**B**) The number of invaded cells was measured via Transwell Invasion Assay. Scale bar = 100 μm (**C**) Western blot analysis of epithelial and mesenchymal markers in GS, GR, and FOXP1 KD GR cells. **D** WST-1 cell viability analysis of GS, GR, and FOXP1 KD GR cells. **E** Immunofluorescence staining of Ki-67 in GS, GR, and FOXP1 KD GR cells. Scale bar = 50 μm (**F**) Western blot analysis of activated MAPK protein expression levels in GS, GR, and FOXP1 KD GR cells. **G** Western blot analysis of expression levels of cell cycle regulators genes in GS, GR, and FOXP1 KD GR cells. Data are represented as the mean ± S.D. of three independent experiments (*n* = 3). Statistical analysis was conducted using one-way or two-way ANOVA, followed by Tukey’s multiple-comparison test. **p < 0.01, ***p < 0.0001.

We analyzed the role of FOXP1 in oncogenic properties of chemoresistant PDAC. Many reports have highlighted the role of CSCs in inducing epithelial-to-mesenchymal transition (EMT) in several tumors [24]. We observed decreased migration of FOXP1 KD cells in a wound healing assay, demonstrating the role of FOXP1 in motility regulation (Fig. 3A). We also found that FOXP1 inhibition led to decreased invasive ability, as KD cells showed reduced invasion in the Transwell invasion assay (Fig. 3B). We then analyzed the gene expression levels of prominent EMT markers, such as E-cadherin, N-cadherin, and Vimentin. The depletion of FOXP1 led to decreased mRNA and protein expression of N-cadherin and vimentin and increased expression of E-cadherin, underscoring the role of FOXP1 in regulating EMT and metastasis in chemoresistant pancreatic cancer (Figs. 3C and S3A). We then analyzed whether the upregulation of FOXP1 leads to increased proliferation of tumor cells. The viability assay revealed an increased proliferation of GR cells, which decreased upon FOXP1 knockdown (Fig. 3D). Additionally, we observed a marked reduction in Ki-67 positive cells in the KD group, reflecting the reduced proliferative ability of FOXP1 depleted cells (Fig. 3E). Therefore, we hypothesized that FOXP1 contributes to increased proliferation of chemoresistant pancreatic tumors. Previous studies on gemcitabine-resistant pancreatic cancers found upregulation of the MAPK pathway in resistant tumors [10, 25]. To determine whether increased FOXP1 expression affected the MAPK pathway, we analyzed protein expression levels in FOXP1 KD cells. We found that both phospho-p38 and phospho-ERK expressions decreased in KD cells (Fig. 3F). The inhibition of FOXP1 led to notable decreases in the expressions of genes associated with cell cycles, such as CDK4/6 and Cyclin D/E (Figs. 3G and S3B). Our findings indicated that FOXP1 drives chemoresistant pancreatic cancer to become more oncogenic, particularly through the enhancement of EMT and increased tumor proliferation.

### FOXP1 induces metabolic reprogramming by promoting glycolysis

**Fig. 4:**
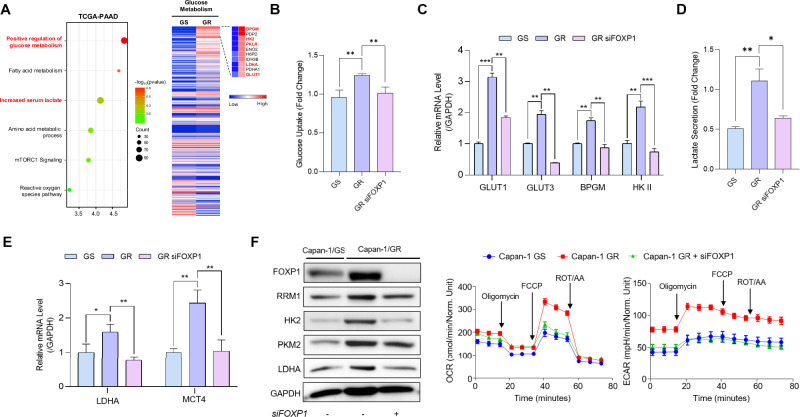
FOXP1 induces metabolic reprogramming by promoting glycolysis. **A** GSEA of the TCGA-PAAD cohort shows that high FOXP1 expression is associated with increased glucose metabolism and serum lactate levels. Heatmap analysis of RNA sequencing data from Capan-1 GS and GR cells revealed the upregulation of glycolysis-related genes in GR cells. **B** Glucose uptake assay showed reduced lactate levels in KD cells compared to GR cells. **C** mRNA expression analysis of glycolytic genes in GS, GR, and FOXP1 KD GR cells, quantified by qRT-PCR. **D** Lactate secretion assay showing reduced lactate levels in KD cells compared with GR cells. **E** mRNA expressions of LDHA and MCT4 in GS, GR, and FOXP1 KD GR cells were quantified by qRT-PCR. **F** Western blotting analysis of glycolytic enzymes in GS, GR, and KD cells. **G** OCR and ECAR assays indicated reduced glycolysis in KD cells. Data are represented as the mean ± S.D. of three independent experiments (*n* = 3). Statistical analyses were conducted using one-way or two-way ANOVA, followed by Tukey’s multiple-comparison test. * *p* < 0.05, ** *p* < 0.01.

CSCs drive metabolic reprogramming in tumors, promoting tumor resistance to chemotherapy. Recent reports found that CSCs promote metabolic reprogramming by upregulating glycolysis [26–28]. A previous analysis of distinct subtypes of pancreatic CSCs found a strong association between stemness and metabolic reprogramming, leading to increased EMT and metastasis [29]. Therefore, we wanted to determine whether increased FOXP1 activity could drive the metabolic reprogramming toward glycolysis in chemoresistant PDAC. We used GSEA to determine whether patient samples with high FOXP1 expressions within TCGA-PAAD cohort had both increased glucose metabolism and serum lactate levels. In addition, RNA sequencing analysis of Capan-1 GS and GR cells revealed that genes associated with glycolysis, including *BPGM, HK*, and *GLUT1*, were upregulated in GR cells (Fig. 4A). To determine whether inhibition of FOXP1 affects glucose metabolism, we first performed glucose uptake assay and found that glucose uptake was significantly decreased in FOXP1 KD cells (Fig. 4B). We also found notable reductions in the expressions of the glucose transporters, GLUT1 and GLUT3, and the glycolytic enzymes, BPGM and HKII, in KD cells (Figs. S4A and 4C). To further determine the effects of FOXP1 on glycolysis, we examined lactate secretion levels and found that lactate secretion was decreased in KD cells (Fig. 4D). Similar to glycolysis, we found downregulation of LDHA and the lactate transporter MCT4 in the KD cells (Fig. 4E). Assessment of protein expression levels also revealed a consistent decrease in the expression of glycolytic and lactate secretion genes (Fig. 4F). Finally, to validate the functional consequences of FOXP1-mediated metabolic reprogramming, we performed oxygen consumption rate (OCR) and extracellular acidification rate (ECAR) assays in GS, GR and KD cells (Fig. 4G). We observed a decrease in both OCR and ECAR in KD cells, indicating that FOXP1 may drive pro-glycolytic pathways in chemoresistant pancreatic cancer.

### FOXP1 chemosensitizes PDAC to gemcitabine by upregulating the expression of ABCG2

**Fig. 5:**
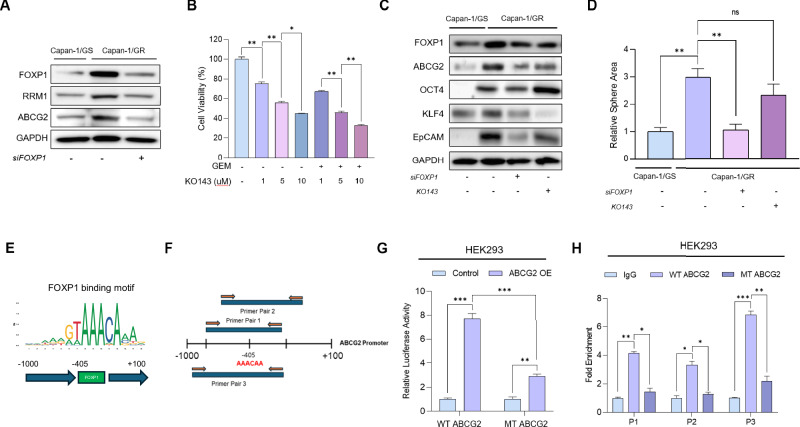
FOXP1 chemosensitizes PDAC to gemcitabine by upregulating ABCG2. **A** Correlation analysis between FOXP1 and ABCG2 expression levels in the TCGA-PAAD dataset via TIMER 2.0. **B** Western Blot shows reduced ABCG2 expression in FOXP1 KD cells. **C** WST-1 viability assay of GR cells treated with KO143 at different concentrations. Co-treatment with gemcitabine and KO143 further decreases cell viability. **D** Sphere formation assay of GS, GR, FOXP1 KD, KO143-treated GR cells, and co-treatment of KO143 and siFOXP1. **E** FOXP1 binding motif and schematic of the ABCG2 promoter region showing potential FOXP1 binding sites identified via JASPAR. **F** Primer design for assessing FOXP1 binding to the ABCG2 promoter. **G** Luciferase reporter assay in HEK293 cells transfected with wild-type (WT) or mutant (MT) ABCG2 promoter constructs. **H** Chromatin immunoprecipitation (ChIP) assay in HEK293 cells confirms FOXP1 binding to the ABCG2 promoter. Data are represented as the mean ± S.D. of three independent experiments (*n* = 3). Statistical analysis was conducted using one-way or two-way ANOVA, followed by Tukey’s multiple-comparison test. *ns* > *0.05*, *p < 0.05, **p < 0.01, ***p < 0.0001.

One mechanism by which CSCs induce chemoresistance in tumors is the upregulation of ATP-binding cassette (ABC) transporters, which induce drug efflux [30]. Although FOX family genes and ABC transporters are correlated [17, 31], there are no previous reports linking FOXP1 to ABC transporters in pancreatic cancer. Therefore, we sought to identify the ABC transporters that were upregulated in GR Capan-1 cells. RNA sequencing analysis of Capan-1 GS and GR cells revealed that ABCG2 was the most upregulated gene among the ABC transporter family members in GR cells (Fig. S5A). We also found a strong positive correlation between FOXP1 and ABCG2 using the TCGA-PAAD dataset (Fig. S5B), suggesting a possible mechanistic regulation of ABCG2 by FOXP1. Silencing of FOXP1 led to a decrease in the expression of ABCG2 and RRM1 (Fig. 5A). We then used KO143, a selective inhibitor of ABCG2 [32], to determine the effects of ABCG2 inhibition on GR PDAC cells. Treatment of KO143 in GR Capan-1 cells led to reduced viability in a concentration-dependent manner. Moreover, the co-treatment with KO143 and gemcitabine significantly reduced cell viability, highlighting ABCG2 inhibition as a potential target for chemosensitizing resistant PDAC cells (Fig. 5C). Studies have revealed that KO143 is a potential inhibitor of CSCs in prostate and liver cancer [33, 34]. We found that treatment with KO143 led to a decreased expression of CSC markers in Capan-1 GR cells (Fig. 5C). In addition, KO143 treatment reduced sphere formation of GR cells, although the reduction was not as significant as the knockdown of FOXP1 (Fig. S5B, D). We then used the transcription factor binding profile database JASPAR to analyze possible FOXP1 binding sites within the ABCG2 promoter region (Fig. 5E). We identified a probable binding site for FOXP1 and designed three primers that included this binding site (Fig. S5C, F). HEK293T cells were transfected with two plasmids: (1) a plasmid in which the ABCG2 promoter contained the wild-type (WT) FOXP1 binding site and (2) a plasmid in which the FOXP1 binding site was mutated via deletion (MT) from the promoter (Fig. S5D). We performed both luciferase reporter and ChIP assays to confirm that FOXP1 binds to the ABCG2 promoter, which has not been previously reported in pancreatic cancer (Fig. 5G, H). FOXP1 mediated chemoresistance in pancreatic cancer by directly regulating ABCG2, further validating the crucial role FOXP1 plays in modulating chemoresistance in pancreatic cancer.

### Reduced FOXP1 and ABCG2 expressions lead to reduced tumor growth and increased chemosensitivity in vivo

**Fig. 6:**
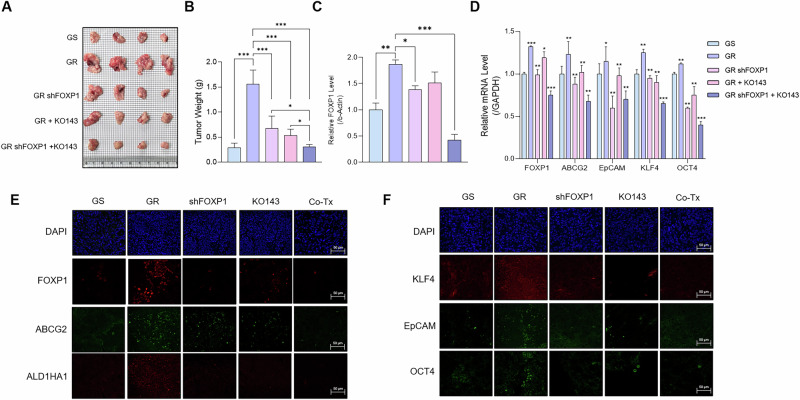
Reduced FOXP1 and ABCG2 expressions lead to reduced tumor growth and increased chemosensitivity in mouse models. **A** Representative image of tumors from BALB/C nude mice injected with GS or GR Capan-1 cells, with GR cells treated with shFOXP1 lentivirus and/or KO143 (Co-Tx). **B** Quantification of tumor weights after 5 weeks shows significantly smaller tumors in the Co-Tx group. **C** Western blot analysis of FOXP1 expressions in GS, GR, shFOXP1-treated, KO143-treated, and Co-Tx groups. **D** Relative mRNA expression levels of stemness markers show significant reductions in the Co-Tx group compared to untreated groups. **E**, **F** Immunofluorescence analyses of FOXP1, ABCG2, and stemness marker genes in tumor tissues from each group. Data are represented as the mean ± S.D. of three independent experiments (*n* = 3). Statistical analysis was conducted using one-way or two-way ANOVA, followed by Tukey’s multiple-comparison test. *p < 0.05, **p < 0.01, ***p < 0.0001.

We assessed the effects of FOXP1 inhibition in vivo using an orthotopic mouse model. 3 × 10^6^ GS and GR Capan-1 cells were injected into randomly selected BALB/c nude mice. Two groups of mice injected with GR cells were further inoculated with shFOXP1 lentiviral particles, and all mice were treated with gemcitabine, KO143, or both gemcitabine and KO143. The mice were euthanized after 5 weeks, and the tumors were harvested (Fig. S6A). We found that the tumor weights of mice treated with both shFOXP1 lentivirus and KO143 (Co-Tx) were significantly lower than those of the untreated groups (Fig. 6A, B). Assessment of FOXP1 protein expression revealed that FOXP1 was the most downregulated in the Co-Tx group, suggesting that the combined inhibition of FOXP1 and treatment with KO143 may not only reduce resistance to gemcitabine but also lead to a decrease in the proliferation of CSCs (Fig. 6C). We examined the mRNA expression levels of stemness-related genes across the groups. The expressions of the stemness markers were reduced in the Co-Tx group, indicating that inhibition of the FOXP1-ABCG2 axis may be crucial in combating chemoresistant pancreatic cancer (Fig. 6D). We further confirmed the changes in gene expression via immunofluorescence, which showed a marked decrease in ABCG2 and ALD1HA1 expression in the Co-Tx group (Fig. 6E). Further imaging showed diminished expressions of stemness markers KLF4, EpCAM, and OCT4 in the Co-Tx group (Fig. 6F). Our results highlight the importance of inhibiting the FOXP1-ABCG2 axis as a therapeutic strategy for combating chemoresistant pancreatic cancer.

## Discussion

Effectively targeting CSCs remains a significant challenge in the development of cancer therapeutics [35]. The rates of recurrence and relapse in pancreatic cancer emphasize the necessity for developing therapeutics that target CSCs [36]. In the present study, FOXP1 is highly upregulated in chemoresistant pancreatic cancer and plays a critical role in upregulating CSC properties and resistance to gemcitabine. We discovered that the inhibition of FOXP1 led to decreased proliferation and increased chemosensitivity via the ablation of ABCG2. Our findings indicate that FOXP1 is a significant biomarker not only for identifying CSCs but also as a potential target for therapeutic strategies against CSCs and chemoresistance.

FOXP1 was previously reported as having both tumor-suppressive and oncogenic roles [37]. In intrahepatic cholangiocarcinoma and prostate cancer, overexpression of FOXP1 was associated with tumor suppression [38, 39]. Notably, even in pancreatic cancer, a report reported that FOXP1 may act as a tumor suppressor [18]. However, despite contradictory reports, our study specifically focuses on the role of FOXP1 in the development and maintenance of chemoresistance. While numerous factors lead to the development of chemoresistance, CSCs have increasingly been the focus of attention [40]. The importance of targeting CSCs in tumors has led to a new perspective on transcription factors [35]. FOXP1 plays a critical role in sustaining stemness in embryonic stem cells and exhibits similar functions in cancer stem cells [41]. FOXP1 also regulates key proliferative pathways, such as STAT3, which enhances tumorigenic potential [15, 42]. Moreover, the inhibition of FOXP1 led to a decrease in the proliferation of cancer stem cells in ovarian cancer [17]. However, the role of FOXP1 in pancreatic cancer, particularly in chemoresistance, has not been explored. Our study demonstrates that FOXP1 is highly upregulated in chemoresistant pancreatic cancer and is correlated with poor patient prognosis (Fig. 1). Our findings suggest that FOXP1 may serve as a prognostic biomarker in patients with chemoresistant pancreatic cancer.

Our study provides compelling evidence for targeting FOXP1 to identify and combat CSCs in chemoresistant cancers. The identification of specific CSC target genes within solid tumors remains a substantial challenge [43]. Although surface markers and stemness-related transcription factors are used as biomarkers for CSCs in malignancies [44, 45], little is known about the mechanisms by which these markers promote and maintain chemoresistance [46]. Our analysis of protein expression in GR spheroids revealed that FOXP1 was highly upregulated in chemoresistant CSCs. The upregulation of genes such as *ALD1HA1*, *OCT4*, *C-MYC*, and *KLF4* clearly indicates the stemness characteristics were increased in the chemoresistant cells (Fig. 2). One interesting observation was the upregulation of *EpCAM* (epithelial cell adhesion molecule) in the gemcitabine-resistant cells. In recent studies, EpCAM expression has been implicated in the induction of the so-called “hybrid EMT,” in which cells exhibit both epithelial and mesenchymal features [47]. Hybrid EMT cells have increased plasticity, which allows cells to survive, metastasize, and evade therapies [48, 49]. Our data reveals that hybrid EMT may induce CSC proliferation and expansion in PDAC, as we found GR cells with high FOXP1 expressions had higher metastatic potential via wound healing and invasion assays (Fig. 3). Our data reveals that inhibiting FOXP1 not only significantly reduces the proliferation of CSCs but also emphasizes its role as a marker of enhanced plasticity and metastasis, which underscores its potential as a valuable translational target.

Furthermore, CSCs have been implicated in promoting metabolic reprogramming that helps contribute to chemoresistance [50, 51]. Glucose metabolism and glycolysis are frequently upregulated in CSC-rich environments [52], which facilitates the reduction of ROS production, allowing cancer cells to survive chemotherapeutic treatments [53, 54]. In PDAC, metabolic reprogramming enhances signaling pathways that lead to the upregulation of stemness-related genes, such as *MYC* [55]. Therefore, CSC-induced metabolic reprogramming is crucial for mediating the survival of chemoresistant tumors. In this study, we found that FOXP1 plays a significant role in modulating the upregulation of glycolysis. Silencing FOXP1 reduced glucose uptake and lactate production and decreased the expression of pro-glycolytic genes. It also reverted the cells to their original metabolic state by decreasing OCR and ECAR, suggesting that FOXP1 may drive metabolic reprogramming in chemoresistant CSCs (Fig. 4).

CSCs also rely on ABC transporters to modulate the efflux of chemotherapeutic drugs, inducing multi-drug resistance in cancers [56, 57]. Although FOXP1 regulates ABC transporters in other cancers [17], there have been no such reports for pancreatic cancer. We found that FOXP1 directly binds to the promoter site of ABCG2 and that inhibiting both FOXP1 and ABCG2 led to increased sensitivity to gemcitabine. Luciferase reporter and ChIP assays using promoter construct plasmids revealed that deletion of the FOXP1 binding site in the ABCG2 promoter decreased ABCG2 expression (Fig. 5). Also, silencing FOXP1 and inhibiting ABCG2 in orthotopic mouse models led to significant reductions in tumor sizes, highlighting the clinical potential of therapeutics against FOXP1 (Fig. 6).

In conclusion, our findings show that FOXP1 plays an essential role in establishing chemoresistance in pancreatic cancer by enhancing CSC characteristics and upregulating ABCG2 expression. Targeting the FOXP1-ABCG2 axis may provide a novel approach to improving patient treatment results. Our findings offer insights into the development of CSC-driven chemoresistance and provide insights into developing more effective treatments.

## Supplementary information

Supplementary Table 1

Supplementary Figures

## Data Availability

The datasets analyzed during the current study are available in the NCBI Gene Expression Omnibus (GEO) repository under accession numbers GSE71989 and GSE28735 and can be accessed via the following links: https://www.ncbi.nlm.nih.gov/geo/query/acc.cgi?acc=GSE71989, https://www.ncbi.nlm.nih.gov/geo/query/acc.cgi?acc=GSE28735.
