# Supplementary material for: The FOXP1-ABCG2 axis promotes the proliferation of cancer stem cells and induces chemoresistance in pancreatic cancer
Source: Cancer Gene Ther. 2025 Apr 1;32(5):563–72. doi: 10.1038/s41417-025-00896-7 (PMC12086089; doi:10.1038/s41417-025-00896-7)
Supplement: Supplementary file 1 — Supplementary Table 1 [file 41417_2025_896_MOESM1_ESM.docx]

# Table 1. Primary Antibody Information

| **Antibody (Clone)** | **Host Species** | **Catalog Number** | **Supplier** | **Dilution** | **Molecular Weight** | **Application** |
| --- | --- | --- | --- | --- | --- | --- |
| FOXP1 (A-2) | Mouse | sc-398811 | Santa Cruz Biotechnology | 1:1,000 | 80 kDa | Western Blot, Immunofluorescence |
| ABCG2 (BXP-21) | Mouse | sc-58222 | Santa Cruz Biotechnology | 1:1,000 | 67 kDa | Western Blot, Immunofluorescence |
| Oct-3/4 (C-10) | Mouse | sc-5279 | Santa Cruz Biotechnology | 1:1,000 | 45 kDa | Western Blot, Immunofluorescence |
| EpCAM (VU1D9) | Mouse | 2929S | Cell Signaling | 1:1,000 | 40 kDa | Western Blot, Immunofluorescence |
| ALD1HA1 (D9Q8E) | Rabbit | 54135S | Cell Signaling | 1:1,000 | 50 kDa | Western Blot, Immunofluorescence |
| KLF4 (D1F2) | Rabbit | 12173S | Cell Signaling | 1:1,000 | 65 kDa | Western Blot, Immunofluorescence |
| GAPDH (G-9) | Mouse | sc-365062 | Santa Cruz Biotechnology | 1:1,000 | 37 kDa | Western Blot |
| Cyclin D1 (H-295) | Rabbit | sc-753 | Santa Cruz Biotechnology | 1:1,000 | 36 kDa | Western Blot |
| CDK6 (C-21) | Rabbit | sc-177 | Santa Cruz Biotechnology | 1:1,000 | 35 kDa | Western Blot |
| CDK4 (C-22) | Rabbit | sc-260 | Santa Cruz Biotechnology | 1:1,000 | 35 kDa | Western Blot |
| Cyclin E (M-20) | Rabbit | sc-481 | Santa Cruz Biotechnology | 1:1,000 | 50 kDa | Western Blot |
| c-MYC (A-14) | Rabbit | sc-789 | Santa Cruz Biotechnology | 1:1,000 | 67 kDa | Western Blot |
| LDHA (E-9) | Mouse | sc-137243 | Santa Cruz Biotechnology | 1:1,000 | 35 kDa | Western Blot |
| HK II (NA.41) | Rabbit | HPA028587-100UL | Sigma | 1:1,000 | 110 kDa | Western Blot |
| RRM1 (EPR8483) | Rabbit | ab137114 | Abcam | 1:1,000 | 90 kDa | Western Blot |
| E-Cadherin (36/E-Cadherin) | Mouse | 610181 | BD Biosciences | 1:1,000 | 120 kDa | Western Blot |
| N-Cadherin (32/N-Cadherin) | Mouse | 610920 | BD Biosciences | 1:1,000 | 130 kDa | Western Blot |
| Vimentin (R28) | Rabbit | 3932S | Cell Signaling | 1:1,000 | 57 kDa | Western Blot |
| PKM2 (D78A4) | Rabbit | 4053S | Cell Signaling | 1:1,000 | 65 kDa | Western Blot |
| p-p38 MAPK Thr180/Tyr182 (D3F9) | Rabbit | 4511T | Cell Signaling | 1:1,000 | 38 kDa | Western Blot |
| p38 MAPK | Rabbit | 9212 | Cell Signaling | 1:1,000 | 38 kDa | Western Blot |
| p-ERK MAPK Thr202/Tyr204 | Rabbit | 9101S | Cell Signaling | 1:1,000 | 44 kDa | Western Blot |
| ERK MAPK (137F5) | Rabbit | 4695S | Cell Signaling | 1:1,000 | 44 kDa | Western Blot |
| CD24 (ML5) | Mouse | 311118 | BioLegend | 1:100 | nan | Flow Cytometry (APC-conjugated) |
| CD44 (BJ18) | Mouse | 338804 | BioLegend | 1:100 | nan | Flow Cytometry (FITC-conjugated) |
