# Supplementary material for: The FOXP1-ABCG2 axis promotes the proliferation of cancer stem cells and induces chemoresistance in pancreatic cancer
Source: Cancer Gene Ther. 2025 Apr 1;32(5):563–72. doi: 10.1038/s41417-025-00896-7 (PMC12086089; doi:10.1038/s41417-025-00896-7)
Supplement: Supplementary file 2 — Supplementary Figures [file 41417_2025_896_MOESM2_ESM.pdf]

Supplementary Figures

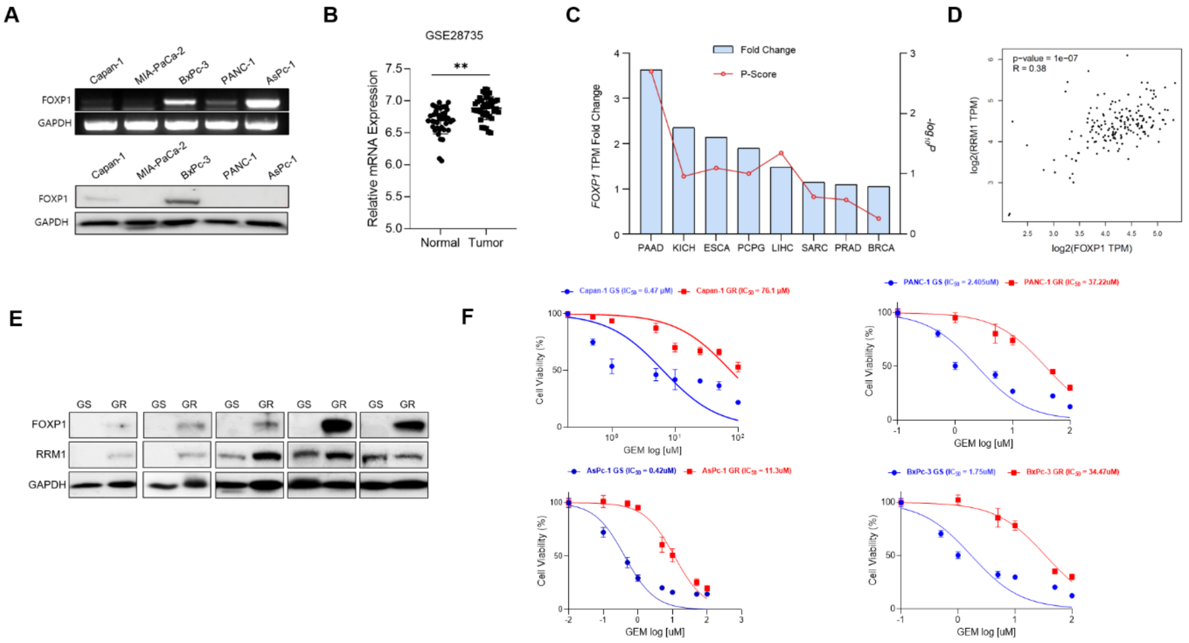

Figure S1. FOXP1 is upregulated in gemcitabine-resistant (GR) PDAC. (A) FOXP1 mRNA and protein expression in PDAC cell lines analyzed via RT-PCR and Western blot. **(B)** Differential gene expression analysis from the GSE28735 dataset reveals higher expression of FOXP1 in tumor samples compared to normal tissues (N=40, T=40) **(C)** Comparative analyses of FOXP1 expression levels across various cancer types (PAAD: Pancreatic Adenocarcinoma; KICH: Kidney Chromophobe; ESCA: Esophageal Carcinoma; PCPG: Pheochromocytoma and Paraganglioma; LIHC: Liver Hepatocellular Carcinoma; SARC: Sarcoma; PRAD: Prostate Adenocarcinoma; BRCA: Breast Cancer) **(D)** Correlation analysis between FOXP1 and RRM1 mRNA levels in the TCGA–PAAD dataset (R = 0.38, p = 1e−07). **(E)** Western blot analysis of FOXP1 and RRM1 protein levels in gemcitabine-sensitive (GS) and gemcitabine-resistant (GR) patient tumor samples. **(F)** Dose-response curves showing the effect of gemcitabine (GEM) treatment on cell viability (%) in GS and GR PDAC cell lines.

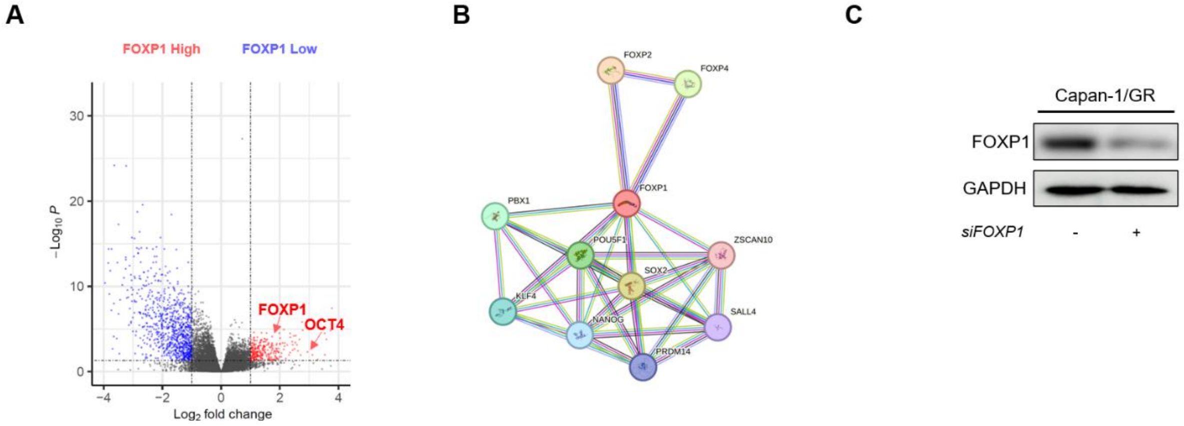

Figure S2. FOXP1 promotes the proliferation of CSCs in GR PDAC. (A) Volcano plot showing differential gene expressions in TCGA-PAAD samples with high versus low FOXP1 expressions. **(B)** STRING protein-protein interaction network analysis of FOXP1. **(C)** The protein expression of FOXP1 after siRNA-mediated knockdown of FOXP1 (siFOXP1).

A

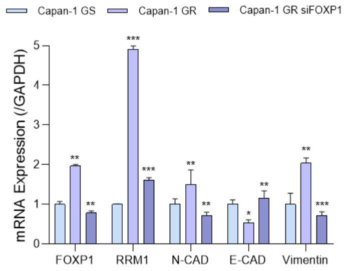

Figure S3. FOXP1 enhances EMT and induces proliferation in GR PDAC. (A) qRT-PCR analysis of mRNA expression levels for FOXP1, RRM1, N-cadherin (N-CAD), E-cadherin (E-CAD), and Vimentin in GS, GR, and KD cells. GAPDH was used as the normalization control. *$p < 0.05$, **$p < 0.01$, ***$p < 0.001$.

A

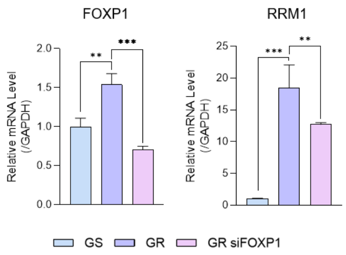

Figure S4. FOXP1 induces metabolic reprogramming by promoting glycolysis. (A) qRT-PCR analysis of mRNA expressions of FOXP1 and RRM1. GAPDH was used as the normalization control. **p < 0.01, ***p < 0.001.

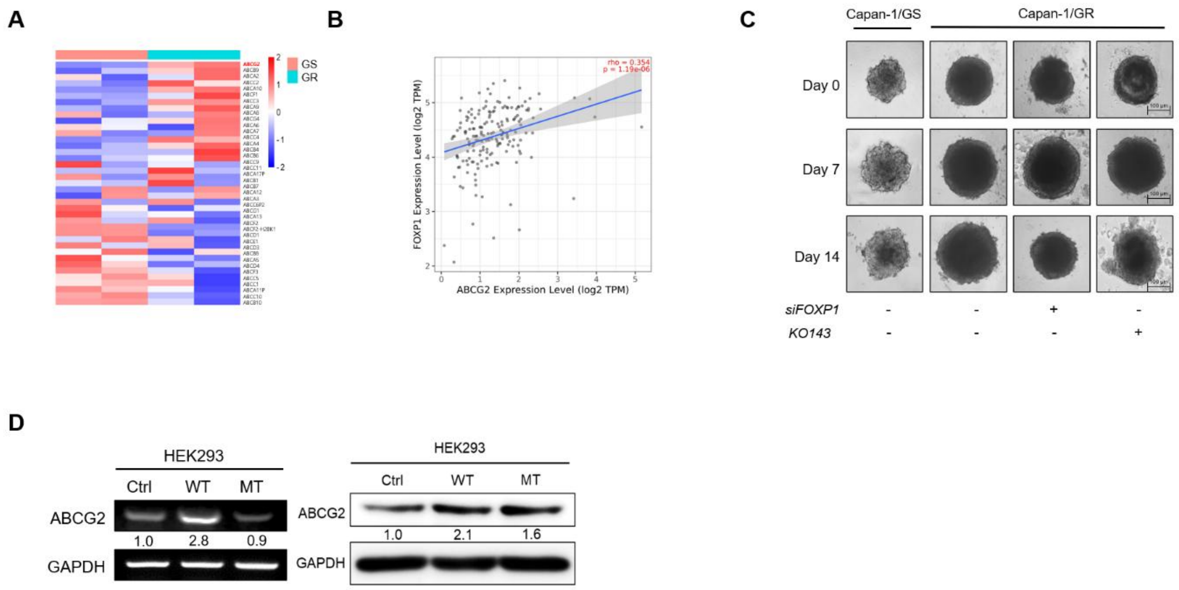

Figure S5. FOXP1 chemosensitizes PDAC to gemcitabine by upregulating ABCG2. (A) Heatmap of ABC transporter gene expression in GS and GR cells. **(B)** Correlation analysis between FOXP1 and ABCG2 mRNA levels in TCGA-PAAD (ρ = 0.354, p = 1.19e−06). **(C)** Relative images of Capan-1 GS and GR spheres treated with siFOXP1 or KO143. **(D)** mRNA and protein expression of ABCG2 in HEK293 cells transfected with wild-type (WT) or mutant (MT) FOXP1.

A

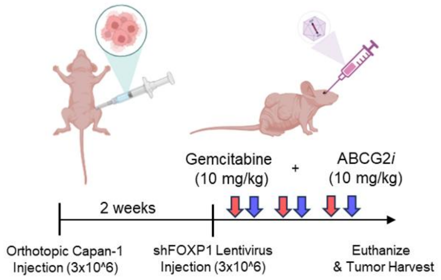

Figure S6. Reduced FOXP1 and ABCG2 expressions lead to reduced tumor growth and increased chemosensitivity in mouse models. (A) Schematic of the orthotopic mouse model. Orthotopic injection of Capan-1 cells was followed by shFOXP1 lentivirus injection after 2 weeks. Gemcitabine (10 mg/kg) and ABCG2 inhibitor (10 mg/kg) treatments were administered prior to tumor harvest.
